# The Role of Serine-Type Serine Repeat Antigen in *Plasmodium yoelii* Blood Stage Development

**DOI:** 10.1371/journal.pone.0060723

**Published:** 2013-04-25

**Authors:** Ximei Huang, Kingsley Liew, Onguma Natalang, Anthony Siau, Neng Zhang, Peter Rainer Preiser

**Affiliations:** School of Biological Sciences, Nanyang Technological University, Singapore, Singapore; Kenya Medical Research Institute - Wellcome Trust Research Programme, Kenya

## Abstract

A key step for the survival of the malaria parasite is the release from and subsequent invasion of erythrocytes by the merozoite. Differences in the efficiency of these two linked processes have a direct impact on overall parasite burden in the host and thereby virulence. A number of parasite proteases have recently been shown to play important roles during both merozoite egress as well as merozoite invasion. The rodent malaria parasite *Plasmodium yoelii* has been extensively used to investigate the mechanisms of parasite virulence in vivo and a number of important proteins have been identified as being key contributors to pathology. Here we have utilized transcriptional comparisons to identify two protease-like SERAs as playing a potential role in virulence. We show that both SERAs are non-essential for blood stage development of the parasite though they provide a subtle but important growth advantage in vivo. In particular SERA2 appears to be an important factor in enabling the parasite to fully utilize the whole age repertoire of circulating erythrocytes. This work for the first time demonstrates the subtle contributions different protease-like SERAs make to provide the parasite with a maximal capacity to successfully maintain an infection in the host.

## Introduction

Malaria is a major public health problem in developing countries. The clinical manifestations associated with malaria infections are caused by the asexual erythrocytic phase of the *Plasmodium* life cycle. A defining feature of malaria infection in human is the multiplication, release and re-invasion of the parasite merozoite into erythrocytes. Within the erythrocyte, parasite undergoes distinct morphological changes from ring to schizont. At the schizont stage, clusters of merozoites are enclosed by a parasitophorous vacuole membrane (PVM) as well as the outer red blood cell membrane. Merozoites are released upon rupture of these two layers of membrane, in an essential process named egress, to invade a new erythrocyte [1]. However, despite the importance of merozoite egress for disease progression, the mechanisms of merozoite release and the molecules involved in the release are largely unknown.

Studies using board-spectrum protease inhibitors have strongly implicated that malaria parasite proteases play crucial roles in parasite infection and development, especially during parasite egress, which has been shown to be a tightly regulated process that involves multiple classes of proteolytic enzymes [2], [3], [4], [5], [6]. Parasite proteases therefore have been considered potential targets for therapeutic interventions. Among all these parasite proteases, two members of subtilisin-like family–SUB1 and SUB2, have been extensively studied and thought to be essential during the blood stage and be involved in host cell invasion [7], [8]. SUB2 has been previously shown to be a sheddase that proteolytically processes the merozoite surface protein 1 (MSP1) as well as apical membrane antigen 1 (AMA-1) both in *Plasmodium falciparum* and *Plasmodium berghei* [9], [10]. More recently, PfSUB1 has also been demonstrated to be involved in the primary processing of the merozoite surface protein complex MSP1/6/7 that primes the merozoite surface for invasion [11]. Besides that, PfSUB1 has also been implicated to regulate parasite egress possibly through direct processing of another group of proteases named serine-repeat antigen (SERA) family [12].

**Figure 1:**
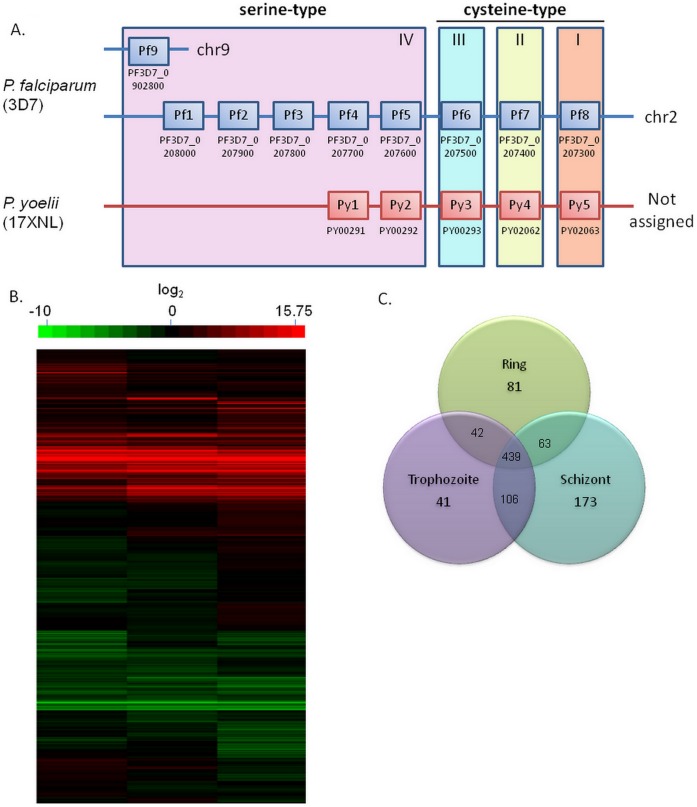
Differences in SERA chromosomal organization and transcription. A. Schematic gene organization of SERA members in *P.falciparum* (3D7) and *P.yoelii* (17XNL). Each rectangular box represents one SERA member and their corresponding gene ID corresponding is indicated below. The horizontal lines represent the chromosomes and confirmed locations of the genes are indicated. SERA members are classified according to the active center residue and monophylic group. Figure is modified from Arisue *et al*., 2007; B. Comparison of YM and YA transcription using microarray analysis. Color scale represents log2 ratio differences between the samples; C. Summary of differentially expressed genes in three different stages. Numbers of genes identified are at each stage or cross stages are indicated.

The SERA multigene family has been identified solely in the genus *Plasmodium* among all the apicoplexan parasites, with the only exception being *Theileria*, a closely related protozoan parasite of cattle [13].The SERAs are highly conserved among the *plasmodium* species, especially in the putative protease domain, suggesting that the functions of these proteases are specific to malaria parasites. *In silico* analysis has identified nine and five members of SERAs respectively in *Plasmodium falciparum* and in the rodent parasite species, *P. yoelii* and *P. berghei* (Fig. 1) [13]. All *Plasmodium* SERAs contain a central, papain-like protease domain and can be classified into two major clusters according to the active site residue, namely cysteine-type SERA and serine-type SERA. One serine-type SERAs–SERA5, as well as one cysteine-type SERA–SERA6, appear to be the most important SERAs in *P. falciparum* as they are expressed at higher levels than most of the other family members and all attempts to disrupt these genes have, to date, been unsuccessful [14], [15]. It has been demonstrated that proteolytic processing of PfSERA5 is associated with schizont rupture and the truncated PfSERA5 product could induce antibodies that either protected against blood-stage infection *in vivo* or interfered with egress or invasion *in vitro* [16], and that high antibody titers of anti-PfSERA5 correlate with protection against severe disease [17]. However, both SERA1 and SERA2 belonging to the serine-type group in *Plasmodium berghei* are not essential in blood stage and knockout of either one of these two SERAs does not affect the normal parasite life cycle [18].

In this study, using comparative transcriptomics we identified two serine-type protease-like antigens (PySERA1 and PySERA2) that are consistently upregulated in a virulent line of *P. yoelii*. The SERA family members in the rodent malaria *P.yoelii* strain have not yet been studied in detail so far. To establish whether these two protease-like antigens have a direct role in parasite virulence, we characterized their role during the asexual blood stage in rodent malaria model *P. yoelii* in more detail. Using genetic modifications, and through loss-of-function study we found that these two SERAs, while not essential for parasite survival in vivo, do provide the wildtype parasite with a growth advantage. Moreover, disruption of PySERA2 attenuates the normally lethal YM strain of *P. yoelii* enabling the mouse to clear the infection. No or only a marginal effect on parasite virulence was observed in the PySERA1 knockout though the parasite did not grow as well as the wildtype parasite in vivo growth competition experiments. Our data suggest that both PySERA1 and PySERA2 while nonessential do provide the parasite with a growth advantage during blood stage growth with the role of PySERA2 being more prominent than PySERA1.

## Materials and Methods

### Parasite Preparation

Male BALB/c mice of 6–8 weeks old were obtained from Sembawang Laboratory Animal Center, National University of Singapore, and subsequently bred under specific pathogen free (SPF) condition at Nanyang Technological University Animal Holding Unit. Mice were infected with cryopreserved stocks of *Plasmodium yoelii* YM strain or YA strain by intraperitoneal injections and parasitemia was monitored by thin blood smears stained with Giemsa.

### Isolation of Different Stages of Parasites

When the parasitaemia reached 40%–60%, mice were terminated and infected blood was collected by cardiac puncture with heparin (Sigma). Parasitized blood was centrifuged at 2100 rpm, 5 min, break 0 at room temperature to remove serum and buffy coat and washed once in incomplete RPMI 1640 (Invitrogen). Different stage parasites (rings, trophozoites and schizonts) were separated and harvested using a 50%–80% Histodenz (Sigma) gradient. Schizonts were cultured till maturity in complete RPMI1640 containing 20% FBS with gentle shaking at 37°C.

### Preparation of Merozoites

ISOPORE membrane filter (Millipore) was pre-wetted with incomplete RPMI1640, and mature schizont culture obtained from above was then passed through the membrane. Free merozoites were then collected, washed once with incomplete RPMI1640 and twice with 1xPBS, and then fast frozen in liquid nitrogen, and stored at a −80 freezer for future use.

### Protein Preparations

Frozen merozoite pellets were disrupted by adding lysis buffer containing 8 M urea, 2 M thiourea, 4% CHAPS and 30 mM Tris (pH 8.0) and three cycles of freezing/thawing in liquid nitrogen and room temperature, respectively. The cells were sonicated on ice over 8 minutes at 30% amplitude. Insoluble material was first pelleted for 60 minutes at 16,100 g at 4°C, and further pelleted for 30 minutes at approximately 100,000 g at 4°C. Proteins were purified by chloroform/methanol precipitation. The protein pellets were air-dried and then resuspended in lysis buffer. Protein concentrations were determined using 2D-Quant Kit from Amersham (GE Healthcare).

### DIGE Labeling

100 µg of each protein preparation was divided into two parts, 50 µg was labeled with 0.4 nM Cy3 and the other 50 µg was labeled with 0.4 nM Cy5 DIGE Minimal Dye Fluors (GE Healthcare) on ice for 30 minutes in dark. Equal amounts of the six samples (3 samples from wildtype YM line and 3 samples from SERA2-KO C1 line) were pooled together as a protein reference pool/internal standard. The protein reference pool was similarly labeled with Cy2. The labeling reactions were stopped by adding 1 µl 10 nM lysine per 0.4 nM CyDye.

### First Dimension: Isoelectric Focusing (IEF)

The isoelectric focusing (IEF) was performed using Immobiline DryStrips (GE Healthcare). For DIGE analysis, 24 cm immobiline strips (pH3–11NL, nonlinear pH gradient) were loaded with 50 µg protein per CyDye (totally 150 µg protein per strip) during rehydration. For protein identification, 24 cm strips were loaded with 500 µg protein per sample. Immobilized pH gradient (IPG) buffer (pH3–11NL; GE Healthcare) was added to a final concentration of 0.5% and 2% to each sample to be loaded on a strip for DIGE analysis and protein identification, respectively, and the total volume of the sample was adjusted to 450 µl by addition of DeStreak Solution (GE Healthcare). Rehydration was carried out more than 12 hours in strip holders placed in the Ettan IPGphor 3 instrument (Amersham, GE Healthcare). IEF was typically carried out in the Ettan IPGphor 3 by holding 0.5 kV for 1 hour, ramping from 0.5 kV to 1 kV over 2 hours, ramping from 1 kV to 8 kV for 3 hours, and holding at 8 kV for 6 hours, yielding a total of about 61 kVh.

### Second Dimension: SDS-PAGE

After IEF was completed, IPG strips were equilibrated under constant agitation in the dark for DIGE analysis and in the light for protein identification at room temperature. They were equilibrated in equilibration buffer (75 mM Tris [pH8.8], 6 M urea, 30% glycerol, 2% SDS) supplemented with 1% (w/v) DTT for 15 minutes first, followed by 15 minutes in equilibration buffer supplemented with 2.5% (w/v) idoacetamide. Strips were briefly washed with 1 × SDS running buffer and then placed on top of the 11% polyacrylamide gels. The gels were run in 1 × SDS running buffer in an Ettan Daltsix Electrophoresis System (Amersham, GE healthcare). Proteins were run until the dye-front reached the bottom of the gels.

### DIGE Data Acquisition and Analysis

Gels after the second-dimension separation were then scanned on a Typhoon Trio scanner (GE Healthcare) at 100 µm resolution. The scanned images were imported into DeCyder 2D software, version 6.5 (GE Healthcare). Spots were detected in the DIA (differential in-gel analysis) module and automatically matched across gels in the BVA (biological variation analysis) module. Spots were either re-matched or excluded manually from the subsequent analysis across all gels. DeCyder 2D software was then used to measure the relative protein abundance. Spot volumes in Cy3 and Cy5 channels were normalized to the corresponding spot volumes in the Cy2 channel. All spot volumes observed in Cy3 or Cy5 channels are multiplied by the corresponding normalization factor. To achieve the spots of interest, the parameters were set as one-way ANOVA p value<0.01, fold change > = 1.4 (up-regulate) or < = −1.4 (down-regulate), spots presenting in > = 18 spots in total images. And fold changes > = 2 or < = −2 are recognized as significant.

### Silver Staining

After the spots of interest obtained, high protein amount (500–800 µg) loaded gels were prepared. The gels were fixed in a solution of 10% acetic acid and 40% ethanol, washed, and sensitized in a solution of 0.2% sodium thiosulfate, 6.8% sodium acetate and 30% ethanol. After washing, the gels were incubated in 0.25% silver nitrate for 5–10 minutes, washed, and developed in a solution of 2.5% sodium carbonate and 0.015% formaldehyde. The staining reaction was stopped in 1.5% Na_2_-EDTA, after further washing, the gels were kept in Milli-Q water at room temperature.

### Gel Spot Identification by MALDI-TOF-TOF Mass Spectrometry

Interested gel spots were excised from the high protein amount loaded gels, and destained in 50 mM Na_2_S_2_O_3_ and 15 mM K_3_Fe(CN)_6_, followed by washes in Milli-Q water and in 100 mM NH_4_HCO_3_. They were dehydrated in a solution of 50% (v/v) acetonitrile and 50 mM NH_4_HCO_3_, and in 100% acentonitrile, and then dried completely in a vacuum centrifuge. They were then digested overnight at 37°C in 12.5 ng/µl trypsin solution (Promega) containing 25 mM NH_4_HCO_3_. The supernatants were collected and digested peptides were further extracted from the gel pieces with 20 mM NH_4_HCO_3_, followed with a solution of 50% (v/v) acentonitrile and 5% (v/v) formic acid. The digested peptides were desalted and concentrated in 0.1% (v/v) trifluoroacetic acid (TFA), in a solution of 50% (v/v) acentonitrile and 0.1% (v/v) TFA using Zip-Tip_µ-C18_ (Millipore), and then dried completely in a vacuum centrifuge. The dried peptides were resuspended in 1 µl of matrix solution containing 10 mg/ml alpha-cyano-4-hydroxycinnamic acid (CHCA) in 50% (v/v) acentonitrile and 0.1% (v/v) TFA and spotted on an Opti-TOF® 384 well MALDI plate (Applied Biosystems). Peptide mass fingerprints and MS/MS fragment ion masses were recorded by MALDI-TOF-TOF mass spectrometry (Matrix-Assisted Laser Desorption/Ionization Time-Of-Flight tandem mass spectrometry) with a 4800 Plus MALDI TOF/TOF Analyzer (Applied Biosystems). Peptide and ion masses were converted into peak lists using Mascot Distiller (Matrix Science) with default parameters. Peak lists were queried against Genbank database (NCBI) using Mascot Server software (Matrix Science).

### RNA Preparation

Pellets of different parasitic stage were mixed with pre-warmed (37^ o^C) Trizol LS (Invitrogen) with immediate vortex. RNA was extracted according to the Trizol LS protocol and then purified using RNA clean-up kit (Qiagen) according to the manufactured protocol. RNA quantitation was done using nano-drop. Purified RNA was performed with DNAse treatment using TURBO DNA-free™ kit (Applied Biosystems Inc), and cDNA was generated by RevertAid™ H Minus M-MiLV reverse transcriptase (Fermentas) or SuperScript® II Reverse Transcriptase (Life Technologies).

### Microarray Hybridization and Analysis

The procedure was done as described previously [19] with modification. Briefly, the cDNA was coupled to Cy3 or Cy5 dye (GE Healthcare) in the presence of 0.1 M NaHCO_3_ (pH9.0) with incubation at room temperature in the dark for 2 hr, and then purified by MinElute PCR purification kit (Qiagen). Both reference (Cy3 labeled) and experimental (Cy5 labeled) samples were mixed with hybridization medium containing 3xSSC, 0.5%SDS and 1 µM HEPES buffer (pH7.5), and then were hybridized to the slides by MAUI hybridization system (BioMicro) at 65°C for 16 hr. Arrays were washed in 2xSSC/0.2% SDS and then 0.1xSSC at room temperature, and then scanned with a GENEPix 4000B scanner and the images were analyzed using GenePix Pro 3.0 software (Axon Instruments). The subsequent data was normalized using NOMAD (http://sourceforge.net/projects/ucsf-nomad/).

### Parasite Transfection

Matured YM schizonts were transfected with linearized construct containing gene of interest and the drug selectable marker using the Basic Parasite nucleofector solution kit II (Lonza) with Amaxa electroporator and the published protocols [20], [21]. Transfected parasites were then introduced into new BALB/c mice by intravenous injection and tranfectants were selected with pyrimethamine (Sigma). SERA1 or SERA2 knockout parasites were firstly obtained by FACs sorting for the GFP signal. Sorted parasites were diluted to single parasite and injected back to BALB/c mice. Obtained parasite lines were then checked and confirmed for the knockout using southern blot.

### Southern Blot Analysis

Genomic DNA was isolated from parasitized erythrocytes using genomic DNA purification kit (Fermentas). Various restriction enzymes were used to digest the gDNA and also disruption plasmids which serve as control. Digested samples were resolved by electrophoresis on a 0.8% agarose gel and transferred onto Hybond N+ membrane (Amersham) in 10xSSC buffer overnight. The membrane was then UV cross-linked prior to hybridization. DNA probes used were partial sequences of the first fragment cloned into the disruption plasmids respectively, using the PCR primer pairs indicated in Table S1, with incorporation of DIG-dUTPs. AP conjugated anti-DIG Ab and CSPD substrate (Roche) was then used and the membranes were scanned using the Typhoon Scanner (GE Healthcare).

### Parasitaemia Growth Curve

To assess the parasite virulence, 5 mice as a group were each injected intravenously with 10^3^ mature schizonts of either wildtype YM or knockout parasite line, and parasitemias were monitored daily using thin blood films stained with Giemsa (Sigma) from day 3 post-infection.

### Selective Index (SI) Determination

Selectivity index as a parameter to determine the selectivity of a parasite to multiply invade a red blood cell, is a well established concept that was first introduced by Simpson, J.A. *et al* [22] and has been previously described in some other studies [23], [24]. Selectivity index was calculated by dividing the observed number of multiple invasions over the expected number of multiple invasions in parasitized red blood cells. For SI determination, parasitaemias were counted using Giemsa stained thin blood films of 3%–13% parasitaemia [22].

### Immunofluorescence Microscopy

Isolation of schizonts was carried out as described before. For immunofluorescence assays, the schizont pellet was resuspended in small volume of iRPMI and smears were then made. The glass slides with the smears were air dried and stored at −20**°**C for future use. Slides were thawed and warmed up to room temperature, then fixed in ice-cold methanol-acetone (1∶1) mix for 5 min. Air-dried slides after fixation, were pre-incubated with 3% BSA at 37**°**C for 1 hr, then incubated with rabbit anti-GFP (Abcam) and mouse anti- py-hep17 (named NYLS3) at 37**°**C for 1 hr. Later they were incubated with Alexa fluor-594 conjugated goat anti-mouse IgG(H+L) and Alexa fluor-488 conjugated goat anti-rabbit IgG(H+L) (Jackson ImmunoResearch) at 37**°**C for 1 hr in dark. Parasite nuclei were stained with DAPI. Washes were done between two antibody incubations and after DAPI for 4 times, 5 min each with 1× PBS. Slides were viewed under Olympus fluorescence microscope at 100× magnification by adding mounting medium for Fluorescence (Vector Laboratories, Burlingame, CA).

### Real-time PCR

Unique primers were designed for 5 members of the SERA genes to amplify short regions (Table 1). Genomic DNA extracted from YM infected blood using the Easy-DNA kit (Invitrogen) was used as an internal standard to compare the primer pair efficiency. A group of three animals were infected with YM or A1–291 C6 line or A1–292 C1 line. RNA extraction and cDNA generation of individual sample was done as described above. Both cDNA samples and genomic DNA samples were amplified with SsoFast™ EvaGreen supermix (Biorad) and analysed on ABI 7000 thermocycler. The relative amount of each member was normalized by actin in individual sample and the relative ratios of each SERA member to actin were calculated.

**Table 1:** Real-time RT-PCR primers for determining transcription of SERA genes.

Real-time PCR Primer Name	Primer Sequence
291 RT f	AATGTTACAAATGTGCATTG
291RT r	AAATATATCTTCAACATCACCT
292RT f	AAATGTGACAAAATAGCTACCAAATG
292RT r	CATCACTTTGTCCAACTGCTAATAC
293RT f	TTGATAACAATGGATGATTTTGATG
293RT r	TTCATTACCTAATTCAGCATTTCC
2062RT f	AATAACGAAATTGACGATGAAAATG
2062RT r	AAAGTGCTGAGCTTCTATAATGACC
2063RT f	TACGAAATAGTTGGGGTTCTAGATG
2063RT r	ATTTGGTGGGTCTACTATCTTAGGG
Actin f	GGTGCTCCAGAAGAACATCC
Actin r	TGGAACAGTATGGGAAACACC

### Competition Assay

Schizonts of YM or SERA1-ko C6 or SERA2-ko C1 were prepared as mentioned above. Two of these parasite lines (YM & SERA1-ko C6, YM & SERA2-ko C1 and SERA1-ko C6 & SERA2-ko C1 respectively) with 10^4^ schizonts each were mixed together and injected intravenously into a group of 4 mice. Parasitemia was monitored daily, and the gDNA was extracted from the parasite mix on day 0 as well as from the tail blood of the infected mice from day 3 post-infection by prepGEM (ZyGEM).. Extracted gDNA was used to quantify the ratio of two different parasite lines with specific unique primers (Table 2) by real-time PCR using SsoFast EvaGreen supermix (Biorad), and analysed on ABI 7000 thermocycler (Applied Biosystems Inc). The respective gDNA of single parasite line (YM or SERA1-ko C6 or SERA2-ko C1) was used as standard to compare the primer pair efficiency.

**Table 2:** Specific primer pairs used in real-time PCR to quality the respectively ratio of parasite lines in three groups of growth competition.

Competition pair		Forward primer	Reverse primer	Length
YM vs SERA1-ko C6	YM-specific	CCATCCGCAGAATCAGTACCAAG	GGAACATCATTAGTTGTGCCATTAA	230 bp
	SERA1-ko specific	CAAGAGTGCCATGCCCGAAG	CATTCTTTTGTTTGTCTGCCATG	217 bp
YM vs SERA2-ko C1	YM-specific	AGATGGTAATACAGTTAGTGGTTTAGGTG	AAAGCAGATGTAGTAAAATTATCATGTCC	226 bp
	SERA2-ko specific	CAAGAGTGCCATGCCCGAAG	CATTCTTTTGTTTGTCTGCCATG	217 bp
SERA1-ko C6 vs SERA2-ko C1	SERA2-ko specific	CCATCCGCAGAATCAGTACCAAG	GGAACATCATTAGTTGTGCCATTAA	230 bp
	SERA1-ko specific	AGATGGTAATACAGTTAGTGGTTTAGGTG	AAAGCAGATGTAGTAAAATTATCATGTCC	226 bp

### Generation of Anti-SERA2 Antibodies

Peptide AAQGQVANGQTGP towards the C-terminal sequence of pySERA2 was obtained from the core facility of School of Biological Sciences, Nanyang Technological University. Antibodies against this peptide were generated in rabbits by i-DNA Biotechnology Pte Ltd.

### Western Blot Analysis

Protein extract of different parasite lines were prepared using laemmli sample buffer (Sigma) and heated at 100°C for 5 min then stored at −20°C for future use. 12% SDS-PAGE was used to separate the parasite extract. The blot was incubated with blocking buffer (Odyssey) at room temperature for 1 hr and then followed by incubation at 4°C overnight with mouse anti-hsp70 (1∶2000 in blocking buffer) and rabbit anti-SERA2 peptide antibody 4209 (1∶1000 in blocking buffer). After washing with 0.1% PBST 4 times 5 min each, the blot was incubated with Alexa fluor-649 conjugated goat anti-mouse IgG(H+L) and Alexa fluor-488 conjugated goat anti-rabbit IgG(H+L) (Jackson ImmunoResearch) at room temperature for 1 hr. In the case of detecting the GFP in addition, the blot was also incubated with chicken anti-GFP (Abcam, 1∶2000 in blocking buffer) primarily besides anti-hsp70 and peptide antibody 4209, and then incubated with Alexa fluor-488 conjugated goat anti-chicken IgG(H+L), Alexa fluor-549 conjugated goat anti-mouse IgG(H+L) and Alexa fluor-649 conjugated goat anti-rabbit IgG(H+L). The blot was washed again with 0.1% PBST 4 times and then once with PBS, 5 min for each wash, then it was dried completely and scanned with the Typhoon Scanner (GE Healthcare) using two or three different channels. And scanned images were analyzed using ImageQuant TL software (Amersham, GE Healthcare).

### Statistics Analysis

Statistical significance was determined with SPSS software. Differences between two experimental groups were analyzed for statistical significance by means of nonparametric Mann-Whitney U test, and for multiple groups Kruskal-Wallis test was used, and un-paired 2-tailed T-test was used to determine the significance of the differences of SERA transcripts. * *p*-value<0.05 or ** *p*-value<0.01 were considered statistically significant.

### Ethics Statement

This study was carried out in strict accordance with the recommendations of the NACLAR (National Advisory Committee for Laboratory Animal Research) guidelines under the Animal & Birds (Care and Use of Animals for Scientific Purposes) Rules of Singapore. The protocol was approved by the by the Institutional Animal Care and Use Committee (IACUC) of the Nanyang Technological University of Singapore (Approval number: ARFSBS/NIE A002). All efforts were made to minimize the suffering.

## Results

### Two SERA Proteases are Transcriptionally Upregulated in Virulent Lines of *P. yoelii*

Microarray data comparing the transcription profile between *P. yoelii* lethal strain YM and the nonlethal strain YA revealed that transcriptional activity could be detected for 5097 out of the 5905 genes present on the array in ring, trophozoite and schizont stages (Fig 1B and Dataset S1). Of these 945 genes showed differential expression with 439 genes found to be differentially expressed (at least 2X change) in all stages (Fig 1C and Dataset S2 and S3). Of these 439 genes approximately 200 genes are annotated with no known function, while another ∼ 180 genes represent *pir* genes. A relatively small number of proteins directly implicated in playing a role in invasion showed differential transcription, including 3 members of the Py235 rhoptry protein as well as MSP8. The differential expression of these merozoite proteins is consistent with previous work that showed that differential expression of Py235 is a contributing factor to parasite virulence [25] while immunization studies with MSP8 have indicated a role of this protein in host cell tropism of the parasite [26]. In addition, two SERA proteases–SERA1 (PY00291) and SERA2 (PY00292) (Fig 1A), previously recognized as SERA3 and a putative cysteine protease respectively, showed some of the biggest differences between the virulent and avirulent parasite lines, with both genes showing a gradual over-expression as the parasite matures. The data was highly reproducible across duplicate experiments (Pearson correlation of 0.953) and we therefore attempted to identify the potential involvement of these two SERA in parasite virulence.

### Localization of the Two SERA Proteases

**Figure 2:**
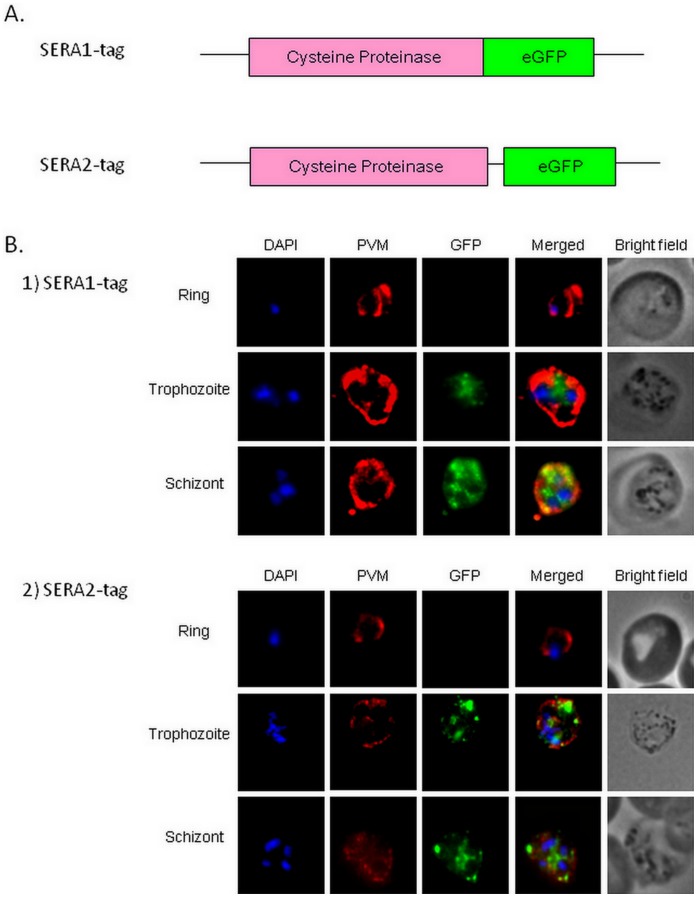
Localization of eGFP-tagged SERA1 and SERA2. A. Schematic representation of constructs of eGFP-tagged SERA1 and SERA2; B. Immunofluorescent assays of parasites with eGFP-tag stably integrated in either SERA1 (upper panel) or SERA2 (lower panel).

**Figure 3:**
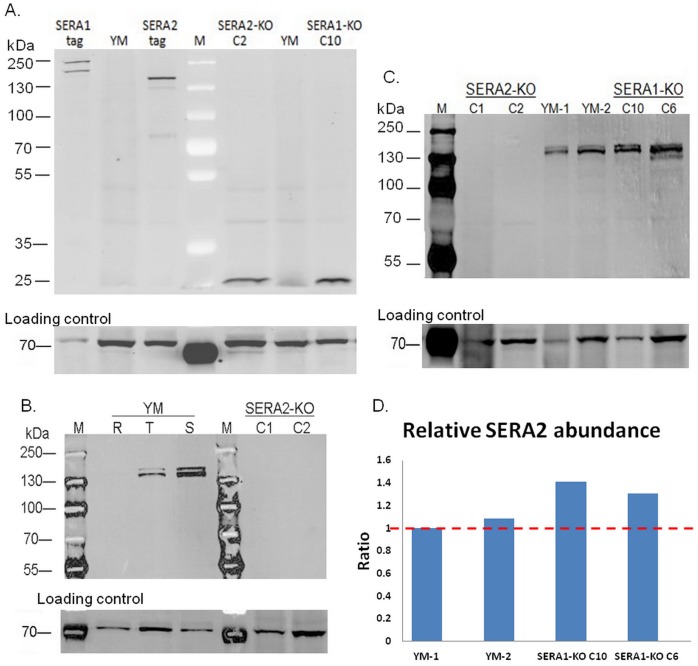
Western blot analysis using peptide antibody against SERA2. A. Presence of GFP in different parasite schizont extract (YM, SERA1-tag, SERA2-tag, SERA1-ko C10 and SERA2-ko C2 lines) was detected using primary chicken anti-GFP antibody and secondary 488@goat anti-chicken.IgG (H+L); while presence of Hsp70 in the same membrane was detected by primary anti-Hsp70 and secondary 549@goat anti-mouse lgG (H+L) as loading control at the lower panel. B. Presence of SERA2 in different parasite extract (YM: ring extract, trophozoite extract and schizont extract; SERA2-ko: schizont extract of C1 line and C2 line) was detected using primary peptide antibody 4209 and secondary 649@goat anti-rabbit IgG (H+L); while presence of Hsp70 in the same membrane was detected by primary anti-Hsp70 and secondary 488@goat anti-mouse lgG as loading control at the lower panel. C. Presence of SERA2 in different parasite schizont extract (YM, SERA2-ko C1 and C2 lines, and SERA1-ko C10 and C6 lines) was detected using primary peptide antibody 4209 and secondary 649@goat anti-rabbit IgG (H+L); while presence of Hsp70 in the same membrane was detected by primary anti-Hsp70 and secondary 549@goat anti-mouse lgG as loading control at the lower panel. D. Band intensities corresponding to SERA2 and Hsp70 in the western blot shown above (Fig 3C) were measured, and the median intensities for bands corresponding to Hsp70 were used for normalization in individual sample to obtain the relative SERA2 expression, and then the relative SERA2 expression ratio was calculated with one of the YM sample as the reference.

To further investigate the role of PySERA1 and 2 in virulence we first accessed their cellular localization. To achieve this both SERA proteases were directly tagged with an eGFP tag that was fused towards the C-terminal end of either SERA gene respectively (Fig 2A and Table S2 and Fig S1) in the YM stain, to create stable parasite lines with eGFP-tagged SERA1 or SERA2. The localization of the GFP was examined under fluorescent microscope in ring, trophozoite and schizont stages (Fig 2B) and showed that the tag (green) remains within the boundaries of the PV (red) but is clearly distinct from the nucleus (blue). Based on the staining pattern it appears that both PySERA1 and 2 are expressed in the later stage of parasite maturation and that the proteases are located insight the parasitophorous vacuole. However, it cannot be ruled out that the observed location in the PV is either due to the GFP tag preventing proper export or due to the fact that SERA are processed at the C-terminal end prior to activation which could result in the GFP tag remaining within the PV while the protease is exported further. However, no obvious free GFP was detected in the parasite schizont extract using anti-GFP antibody (Fig 3A) in SERA1-tag and SERA2-tag as well as YM samples, as compared to the SERA knockout samples which contain the free GFP and showed a band at around 27kDa.

To confirm the timing of expression observed with the GFP tag, the expression of PySERA2 was assessed using western blot with a peptide antibody generated against a region near the C-terminal of the protein. Antibody against Hsp70 was used as a loading control (Fig 3B and C), and gave the expected band of around 70 kDa for each lane. The anti-SERA2 peptide antibody detected bands around 140 kDa in YM trophozoite as well as schizont extract with comparatively higher expression in schizont stage. No product was detected in the ring stage consistent with the GFP expression pattern seen in the IFA and shows that these SERA are prominently expressed in the late stage of parasite development. While the detected band on the western blot appears to be slightly larger than the predicted size of PySERA2 (123.7 kDa), specificity of the antisera was validated using the PySERA2 knock-out parasite (Fig 3B and C and see below).

### Targeted Disruption of the SERAs

To further investigate the potential functions of these two SERAs in the asexual blood stage, we generated loss-of-function parasite lines by disrupting most of the open reading frame with an insertion plasmid through double cross-over homologous recombination (Fig S2). Transfected parasites were first selected through FACs sorting for the green fluorescent signal, and sorted parasites were subsequently diluted for cloning. Successful integration of the plasmid into the SERA1 locus can be detected by Southern blot analysis of SacI digested DNA, with a 4 kb band indicating the correct integration while a fragment of 6.37 kb represents the original locus. The targeting vector that has not integrated would be detected as an approximately 6 kb band on the blot. In the case of SERA2, successful integration of the plasmid into the locus can be detected at around 3.7 kb of SacI/ScaI digested DNA and the original locus would be detected at 3.3 kb, while the non-integrated plasmid should show a band of 5.3 kb on the blot. Several attempts to disrupt both SERA1 and SERA2 simultaneously have not been successful (data not shown).

### Disruption of SERA2 Affects Parasite Growth and Host Survival in Balb/C Mice

**Figure 4:**
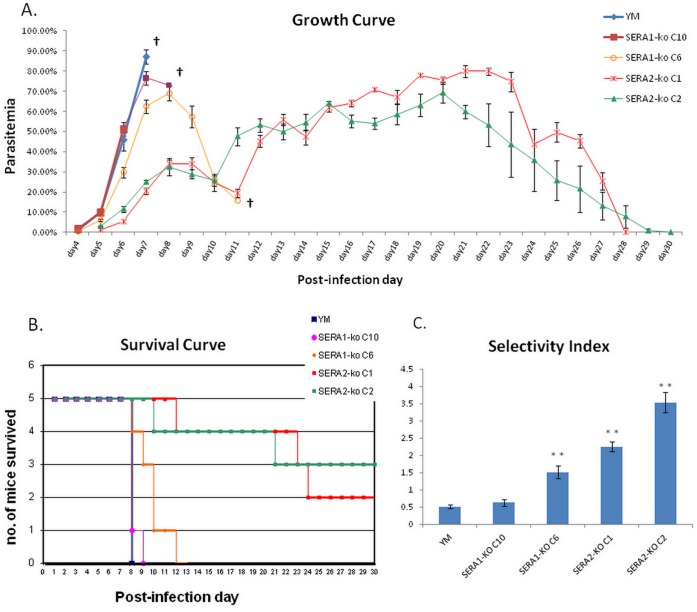
Comparison of growth characteristics between wildtype YM and SERA knock-out lines. A. Parasitemia of Balb/C mice with 10^3^ parasites injected by iv. on day 0 and recorded daily. The average parasitemia of 5 mice for each group were presented.† denotes the death of animals; B. Survival of Balb/C mice infected with YM or KO lines. Note that there was one outlier mouse infected with SERA1-ko C6 survived beyond post-infection day9 but ultimately died two days later; C. Average selective index of 5 BALB/c mice infected with YM or KO lines. Parasite smears were analyzed when parasitaemia was in the range of 3–13%. Differences in SI between YM and SERA1-ko C6 and SERA2-ko C1 and C2 were significant (p<0.01), indicated by **.

While we were unable to generate an antibody against PySERA1, the peptide antibody generated against PySERA2 was able to validate the knockout by allowing us to directly demonstrate the complete absence of SERA2 expression in the C1 and C2 clones (Fig 3B and C). To access the impact of disruption of SERA1 and SERA2 on parasite virulence, 5 mice were infected with 10^3^ parasites of wildtype virulent strain YM, SERA1-ko C6, SERA1-ko C10, SERA2-ko C1 and SERA2-ko C2 respectively. The parasitemia was monitored each day after infection (Fig 4A), and the survival of host mice was calculated (Fig 4B). In the virulent strain YM, parasitemia rose rapidly and all mice died on day 7 post infection with a peak parasitemia >80%. For the SERA1-ko C10 line, parasitemia increased similarly to that of YM and there was no significance difference (*p-value*>0.1), and 4/5 mice died on day 7 with a peak parasitemia >70% while the remaining one mouse died on day 8. In the SERA1-ko C6 line, parasitemia went up less rapidly (*p-value*<0.05) and the peak appeared at around 70% on day 8, 4/5 mice died after that, and the remaining one survived for another two days. In contrast for the SERA2 knockout lines, parasitemia increase in C1 infected mice was significantly slower (*p-value*<0.01), and infected mice survived much longer with only one mouse dying before day 12 and two mice dying after the peak of the parasitemia ∼80% on day 21 and day 22. 2/5 mice infected with C1 eventually survived and parasites were cleared. Mice infected with C2 showed similar pattern, with a significant difference in parasitemia development (p-value = 0.008) and 3/5 mice surviving the infection and the peak parasitemia reaching around 70%.

Selectivity Index (SI) has been previously shown to link to the parasite virulence. Though there is no linear relationship, the lower the SI the more likely that the parasite is virulent and the less likely the mouse host will survive in the infection in *P.yoelii* model [25]. The SI was therefore determined for all mice at a parasitemia of between 3% to 13% (Fig 4C). There are significant differences in the average SI between wildtype YM and SERA2-ko C1 and C2 (p-value <0.01). For the SERA1-ko lines, the average SI for C10 is ∼0.6 which is not significantly different from that of YM (p-value = 0.222), in an agreement with the no significant difference in the parasitemia and host survival as compared to YM. While the C6 line with an average SI ∼1.5 is significantly different from that of YM (p-value <0.01) consistent with the longer survival of the mice infected with this parasite.

**Figure 5:**
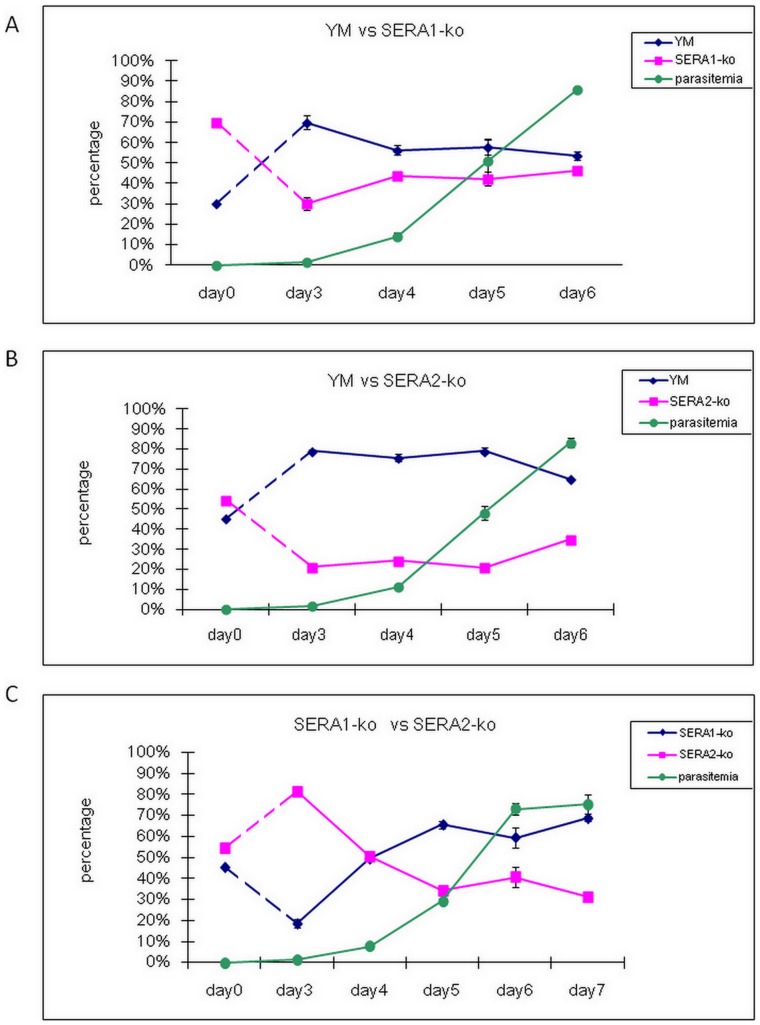
Growth competition between two different parasite lines. Parasitemia of Balb/C mice with 10^4^ parasites of each parasite line were mixed and injected by iv. on day 0 and recorded daily. The average parasitemia and percentage of gene expression calculated through real-time PCR of 4 mice in each group were presented. A. growth competition of YM and SERA1-ko; B. growth competition of YM and SERA2-ko; C. growth competition of SERA1-ko and SERA2-ko.

To further investigate the impact of the SERAs disruption in parasite growth, we explored whether the different parasite lines showed different proliferation potential in growth competition assays *in vivo*. For this approximately equal amount of YM vs PySERA1-ko C6, YM vs PySERA2-ko C1 and PySERA1-ko C6 vs PySERA2-ko C1 were mixed together and used to infect mice. The exact parasite ratios for each experiment were determined using real-time PCR at the start of the experiment (day 0) and after parasites were detected in the blood smear (day 3 onwards). In all these assays YM exhibited a growth advantage over both the SERA1-ko as well as SERA2-ko (Fig 5A & B). Interestingly while there was a clear growth advantage for YM during the early stage of the infection the ratio of wildtype to knockout parasite stabilized and remained constant for the remainder of the infection. The ratios at which the parasite ratios stabilized were different: in the case of YM vs PySERA1-ko the ratio being about 55∶45 while in the case of YM vs PySERA2-ko the ratio being 65∶35. This is consistent with the observation that the PySERA2-ko has a greater reduction in virulence as compared to PySERA1-ko. Competition between SERA1-ko and SERA2-ko lines (Fig 5C) showed an initial growth advantage of PySERA2-ko though this seems to be reversed by about day 4 when PySERA1-ko starts to outgrow PySERA2-ko with the ratio finally stabilizing around 70∶30.

### Impacts of SERA1 or SERA2 Disruption on the Expression Patterns of other SERAs

**Figure 6:**
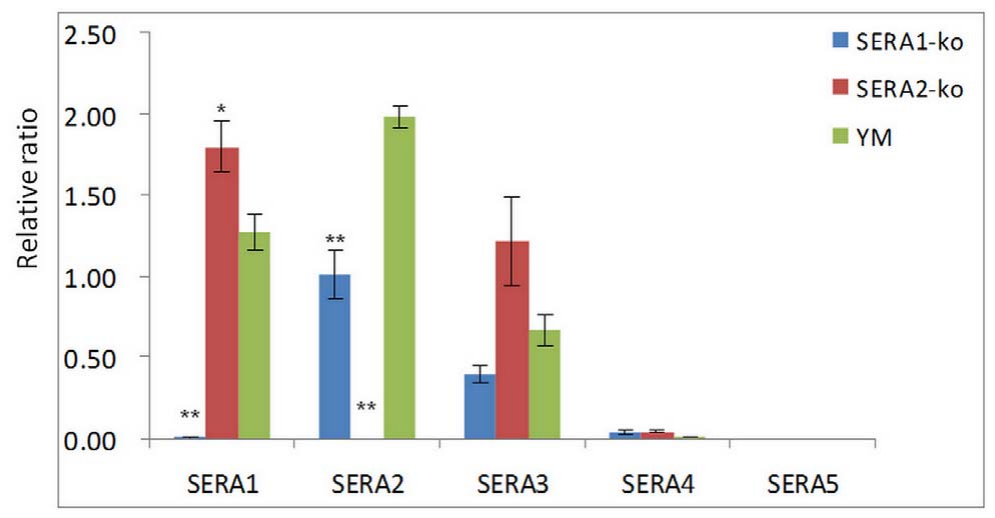
Relative ratio of different SERA members transcribed in three parasite strains. Transcription of individual SERA member was detected using gene-specific primer pair to amplifiy cDNA, and normalized to actin transcription level. Difference in the relative ratio was analyzed for statistical significance between knockout lines (either SERA1-ko or SERA2-ko) and wildtype YM.

To access whether disruption of SERA1 or 2 was compensated by the increased expression of other SERA members, real-time RT-PCR was performed and percentages of the five known SERA members were compared at the transcriptional level between the unmodified YM strain and the two SERA knockout strains (Fig 6). The transcription of both SERA4 and SERA5 was extremely low in all the parasite lines analyzed. Interestingly, disruption of SERA1 led to a significant decrease of SERA2 transcription (p-value = 0.004) while disruption of SERA2 led to a significant increase (p-value<0.05) in SERA1 transcription. However, at protein level, expression of SERA2 at schizont stage showed around 30% increase in SERA1-ko lines as compared to wildtype YM (Fig 3C and D).

### Quantitative Proteomics Reveals no Differences in Merozoite Invasion Protein Expression Levels

The differences in parasite virulence observed between different strains of *P. yoelii* are thought to be mainly due to differences in the red blood cell selectivity and therefore invasion efficiency. This suggests that differences in parasite virulence observed in the SERA2 knockout parasite would be in some form reflected in differences in protein expression or processing at the merozoite stage. We therefore investigated whether mature merozoites obtained from either the YM or PySERA2-ko line showed differences in protein expression or processing using 2D-DIGE (Fig S3). The quantification of the protein abundance was done as previously described [27]. Using a 1.4 fold difference in protein expression (*p-* value <0.01) as a cut off enabled us to identify fifteen proteins that showed significant differences between YM and the knockout parasite (Table 3). No difference in any known invasion related proteins can be detected using this approach; instead we observe a striking upregulation of six glycolytic enzymes in the PySERA2-ko while in the YM line one heat shock protein, elongation factor EF-1 subunit alpha, tubulin and actin show increased expression. While this data does not directly indicate a putative target of the SERA2 in *P. yoelii* it does suggest that the change in host cell tropism of the SERA2 knockout, as reflected in the change in the Selectivity Index, requires some changes in parasite metabolism.

**Table 3:** Merozoite proteins identified with significant difference between YM and SERA2-ko line.

Spot No.	Accession	Description	YM/Sera2 KO Average Ratio	Gene
1	gi|68525541	hypoxanthine phosphoribosyltransferase	−1.45	py03478
9	gi|68525541	hypoxanthine phosphoribosyltransferase	−1.44	py03478
5	gi|50400235	Enolase	−1.43	py06644
23	gi|82752500	Enolase	−1.46	py06644
12	gi|81177589	translation elongation factor EF-1, subunit alpha	3.94	py00361
17	gi|81177589	translation elongation factor EF-1, subunit alpha	1.69	py00361
2	gi|83317699	glyceraldehyde-3-phosphate dehydrogenase	−1.48	py03280
4	gi|82541204	phosphoglycerate kinase	−1.42	py04547
7	gi|83317939	malate dehydrogenase	−1.41	py03376
10	gi|83286382	hypothetical protein/GTP-binding protein	−1.47	py02266
11	gi|13877307	fructose 1,6-bisphosphate aldolase	−1.94	py03709
22	gi|83273948	Ribonucleoprotein	−1.55	py01815
29	gi|82594363	heat shock protein	2.51	py05001
30	TIGR|PY02240	Actin	1.64	py02240
35	gi|82596321	tubulin subunit beta	1.57	py05711

Protein spots with selection numbers and the corresponding gene information are indicated and up-regulation (+) or down-regulation (−) in the fold changes of average ratio are presented.

## Discussion

Based on the homology comparison work to identify SERA proteins in the malaria parasite species by Arisue *et al*. [13], all SERA genes are categorized into Groups I to IV according to the gene structure and phylogenetic relatedness, and five putative SERA proteins were identified in *P. yoelii*. Among these, the two serine-type protease-like antigens PySERA1 and PySERA2 were identified based on transcriptional profiling as potential virulence mediators in the present study. These two protease-like antigens have not yet been studied in detail in *P.yoelii* system so far. Our data shows that these two SERAs are present and expressed abundantly during the later part of blood stage development, and GFP-tagged SERA proteins are located predominantly within the PV. Though these two protease-like antigens belong to the same phylogenetic group as PfSERA5 that has been shown to be refractory to genetic disruption, neither PySERA1 nor PySERA2 was shown to be essential for parasite blood stage development. This is similar to what has been observed for SERA1 and SERA2 in *P. berghei* [18]. However, unlike for *P. berghei*, simultaneously knockout of both of the PySERA1 and PySERA2 has not been possible, suggesting an important and potential complementary role of these two SERAs in *P. yoelii* while also highlighting some potential functional differences between these SERAs in *P. berghei*. While there appears to be some transcriptional adjustment in PySERA1 and PySERA2 in the different knockout lines in all cases changes are less than two-fold and it is at this stage difficult to assess whether these represent true biological compensation mechanism or are rather an indirect effect of the genetic modification of the SERA locus. Transcriptional compensation has been seen in *P. falciparum* for the serine-type SERA subfamily, where the knockout of SERA4 led to up-regulation of transcription of SERA5 [15]. The transcriptional adjustment seen in the work here is most likely not able to explain the partial lethal or decreased mortality phenotype in the knockout lines. Importantly based on the transcriptional profiles in *P. yoelii*, of the five known SERA members, PySERA1 and PySERA2 appear to be the major SERAs in parasite blood stage, while PySERA4 and PySERA5 would be expected to play a minor role. At this stage all the data obtained is consistent with the fact that the phenotypic changes observed after the genetic disruption of PySERA1 and PySERA2 are solely due to the disruption of the respective genes. To completely rule out any secondary effect it will be important in the future to carry out complementation experiments for providing definitive proof of the association between gene and phenotype.

To date, PfSERA5 is the most extensively studied member of SERA family. It has been suggested that PfSERA5 mediates parasite egress through subsequent processing of cellular substrates upon cleavage by subtilisin-like serine protease subtilase 1 (PfSUB1) [12] and that it associates with the merozoite surface presumably by interacting with an integral membrane protein [28]. In our study, disruption of PySERA2 led to changes in parasite phenotype as well as host survival. Given that PySERA2 belongs to the same group of serine repeat antigens as PfSERA5, it is not unlikely that PySERA2-ko may directly impact on merozoite protein expression or modification. In addition, the fact that in *P. yoelii* differences in virulence are linked to changes in invasion suggested that PySERA2 might directly impact on the processing or expression of merozoite invasion proteins. However, 2-dimensional gel electrophosis coupled with MS/MS proteomic analysis on merozoite proteins extracted from YM as well as SERA2-ko parasites, did not identify any significant difference in the expression or modification of any invasion related proteins between these two parasite lines. This lack of any difference in the quantitative proteomics analysis may reflect the limitations of 2D-DIGE to detect all proteins or alternatively indicates that the target of PySERA2 is not found in the released merozoite but is rather located in the maturing schizont or the host erythrocyte. However, we also cannot rule out the possibility that instead of dominant differences of certain known surface antigens, there are minor changes in some groups or some types of proteins with which combined effect becomes amplified and thus significant enough to alter the parasite phenotype. Moreover, it raises the question whether this non-conventional serine residue at the active center of the protease domain in this serine-type PySERA2 retains the enzymatic function, though it has been demonstrated that the 50-kDa central protease domain of PfSERA5 still possessed chymotrypsin-like activity in *in vitro* assays [29]. Alternatively, these findings provide additional support to the idea that this serine-type protease-like antigen plays a role in immune evasion rather than parasite invasion, as suggested by Arisue *et al*. recently [30], where serine-type SERAs as well as Group II and III cysteine-type SERAs are proposed as duplicated genes of Group I SERAs during the evolutional development.

One striking observation of the proteomic approach is the upregulation of six glycolytic enzymes in the SERA2 knockout parasite. Previous work has shown that the redox and energy state of erythrocytes changes as the cell matures, with reticulocytes having a higher ATP/ADP and a lower NAD+/NADPH ratio than mature erythrocytes [31]. One attractive hypothesis that could explain this striking upregulation would be that due to the changed host cell tropism of the knockout parasite as reflected by the changed SI, the parasite has to deal with a different metabolic environment and the changes in the expression of the glycolytic enzymes reflect this adaptation process. Whether this is indeed the case in all reticulocyte restricted parasites or reflects a unique adaptation seen in the SERA2 knockout will need to be investigated in the future.

Normal mice have reticulocyte counts of 2%–5% [32]. Therefore, parasite selective index was assessed at an early infection stage where the parasitemia was between 3%–13%, such that mice have similar hematocrits and parasitemia, ensuring that multiple invasions observed reflects the red cell preference rather than a random process, which is an expected consequence of the parasite density when the parasitemia is high [22]. The higher SI of the knockout lines reflects a potential role of SERAs in host cell recognition and binding/adhesion. It has been demonstrated that PfSERA5 exhibits noncatalytic functions [18], [33], [34] that may help in expanding the host cell range. The knockout lines show a more restricted host cell tropism as compared to YM and this can partially explains the phenomena observed in the growth competition between different parasite lines. While during the early stages of the infection, the ability of YM to invade a wider range of erythrocytes would give it a competitive advantage in the later stages of the infection when the pool of available erythrocytes is mainly determined by the ability of the host to produce new erythrocytes. This advantage would be less pronounced resulting in a stabilization of the relative parasite levels. However, the mechanism behind the interesting “stabilization” of the parasite percentage along the time until the host died remains unknown. It is interesting to note that while the knockout lines have a higher SI compared to YM, the peak parasitemia reached during the infection did not appear to be significantly different from that of YM. The key difference in the SERA2-ko lines is therefore not the absolute parasitemia reached but rather the time taken to reach it. While in YM the peak parasitemia is reached by day 6 in the SERA2-ko lines this is not reached until around day 19–20 and this would provide enough time for the host immune system to be activated and respond to parasite infection. Further analysis on host immune response against different parasite lines may help to test this hypothesis.

The work presented here demonstrates so far unknown roles of two protease-like serine repeat antigens in the outcome of an infection *in vivo* in *P.yoelii* model. Importantly, it highlights subtle differences in the knockout parasite that are difficult to assess *in vitro* but have significant impact on the overall pathology of the infection. Unlike in *P. berghei,* PySERA1 and PySERA2 appear to play an overlapping role and PySERA1 and PySERA2 appear to be able to partially compensate for each other *in vivo*. Moreover, the work also identifies so far unseen adjustments in the parasite proteome that enable the parasite to compensate for changes in host cell tropism. Most importantly this work identifies a so far unknown role of SERA proteases in parasite virulence and highlights the importance of these proteins as targets for therapeutic intervention.

## Supporting Information

Figure S1 **Generation of eGFP-tagged parasites.** A- Schematic depicting a single cross-over gene targeting construct strategy. C-terminal fragment of either SERA1 or SERA2 gene was cloned into B3DhRap2/3-eGFP vector (indicated as pink) with the eGFP in frame. Homologous recombination with the linearized plasmid containing the selectable marker hDHFR results in eGFP fused towards the C-terminal of either SERA1 or SERA2 gene respectively, such that the expression of eGFP is controlled by either the SERA1 or SERA2 endogenous promoter. B- PCR screening determined the successfully integrated parasite lines using 5′- and 3′- integration PCR primer pairs. (1) Lanes 1–3 represent the 5′-integration PCR screen for SERA1 of wildtype YM, vector control and transfected parasite DNAs respectively. Transfected parasites showed to be PCR positive with a faint 1.73 kb target band while the wild-type and vector controls were negative. Lanes 4–6 represent the 3′-integration PCR screen for SERA1 of wild-type, vector control and transfected parasite DNAs respectively. Only the transfected parasites were PCR positive, showing a 1.83 kb band. (2) Lanes 1 and 2 representing the 5′- and 3′- integrations respectively of the transfected parasites, showed PCR positive with the target 1.43 kb band and 1.55 kb band, while lanes 3 and 4 with the wildtype YM gDNA were PCR negative with only the primer dimer present on lane 3.
(TIF)

Figure S2 **Disruption of SERA1 or SERA2 using homologous recombination.** A- Genomic locus MALPY00082 coding for SERA1 and SERA2 showing the regions (red and purple in SERA1, orange and blue in SERA2) used for targeting the locus by a double cross-over strategy. Homologous recombination with the linearized plasmid containing the selectable marker and a detection marker flanked by the targeting sequences results in the SERA1-KO locus or SERA2-KO locus. GFP driven by the constitute promoter pbef1 is used for primary selection by FACs sorting. Restriction sites used for Southern blot analysis as well as region used for Southern blot probes (S1 probe and S2 probe) are also indicated. B- Southern blot screening of parasites for correct integration. (1) SacI digested DNA obtained from wild type YM (lane7) and transfection plasmid (lane6) as well as transfected parasite lines by limiting dilution C1 to C10 (lane1–5 and lane8–12) was analyzed by Southern blot using a SERA1 specific probe (S1). The expected fragment of 4 kb can be seen in all obtained transfected parasite lines, C6 and C10 were selected for further analysis.(2) SacI/ScaI digested DNA obtained from YM (lane 3) and tansfection plasmid (lane2) as well as transfected parasite lines by limiting dilution C1 to C4 (lane4–7) was analyzed by Southern blot using a SERA2 specific probe (S2). A single band of the expected fragment of 3.7 kb can be seen in all obtained parasite lines, C1 and C2 were selected for further analysis.
(TIF)

Figure S3 **Representative two-dimensional DIGE gel of** ***P.yoelii*** **merozoite proteins comparison between wildtype YM and SERA2-ko with silver staining.** Reference spots are marked with red circles, and spots for protein of interest selected for MALDI-TOF-TOF mass spectrometry are indicated.
(TIF)

Table S1 **Primer pairs for generating southern blot probes.**
(DOCX)

Table S2 **PCR primers for eGFP-tagged SERA1 and SERA2 parasites determination.**
(DOCX)

Dataset S1 **Transcription profile between** ***P.yoelii*** **YM and YA strains.** This dataset includes the total 5905 genes present on the microarray. Numbers presented here are the log2 ratio between YM and YA strains.
(XLSX)

Dataset S2 **List of genes differentially expressed between** ***P.yoelii*** **YM and YA strains.** This dataset includes the 945 genes. * indicates the presence of the differences in all three asexual blood stages. Numbers presented here are the log2 ratio between YM and YA strains.
(XLSX)

Dataset S3 **List of genes differentially expressed in all three stages in** ***P.yoelii*** **YM and YA strains.** This dataset includes the 439 genes. Numbers presented here are the log2 ratio between YM and YA strains.
(XLSX)
