# Supplementary figures and images for: The Role of Serine-Type Serine Repeat Antigen in Plasmodium yoelii Blood Stage Development
Source: PLoS One. 2013 Apr 25;8(4):e60723. doi: 10.1371/journal.pone.0060723 (PMC3636278; doi:10.1371/journal.pone.0060723)

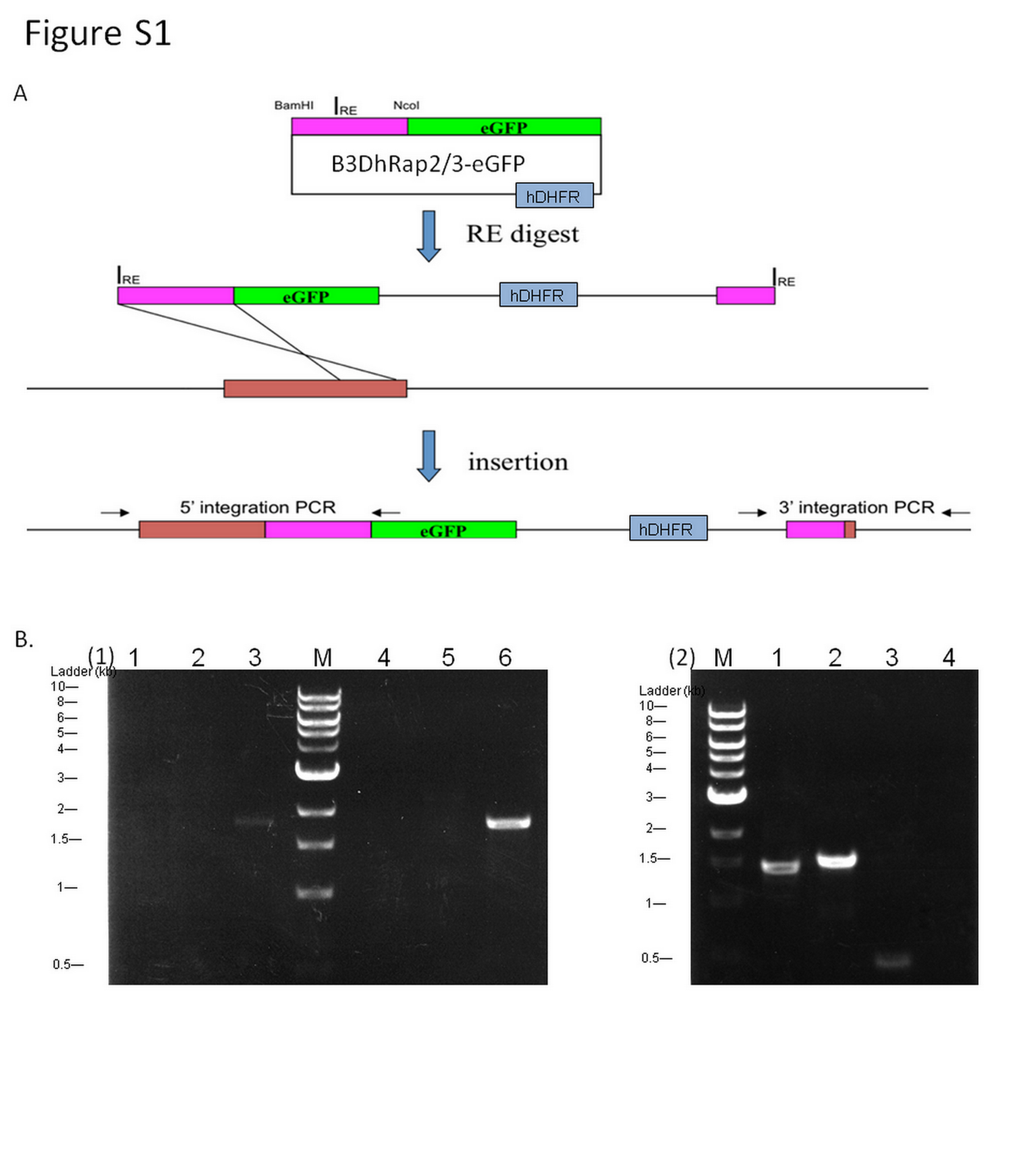

Supplement: Figure S1 — Generation of eGFP-tagged parasites. A- Schematic depicting a single cross-over gene targeting construct strategy. C-terminal fragment of either SERA1 or SERA2 gene was cloned into B3DhRap2/3-eGFP vector (indicated as pink) with the eGFP in frame. Homologous recombination with the linearized plasmid containing the selectable marker hDHFR results in eGFP fused towards the C-terminal of either SERA1 or SERA2 gene respectively, such that the expression of eGFP is controlled by either the SERA1 or SERA2 endogenous promoter. B- PCR screening determined the successfully integrated parasite lines using 5′- and 3′- integration PCR primer pairs. (1) Lanes 1–3 represent the 5′-integration PCR screen for SERA1 of wildtype YM, vector control and transfected parasite DNAs respectively. Transfected parasites showed to be PCR positive with a faint 1.73 kb target band while the wild-type and vector controls were negative. Lanes 4–6 represent the 3′-integration PCR screen for SERA1 of wild-type, vector control and transfected parasite DNAs respectively. Only the transfected parasites were PCR positive, showing a 1.83 kb band. (2) Lanes 1 and 2 representing the 5′- and 3′- integrations respectively of the transfected parasites, showed PCR positive with the target 1.43 kb band and 1.55 kb band, while lanes 3 and 4 with the wildtype YM gDNA were PCR negative with only the primer dimer present on lane 3. (TIF) [file pone.0060723.s001.tif]

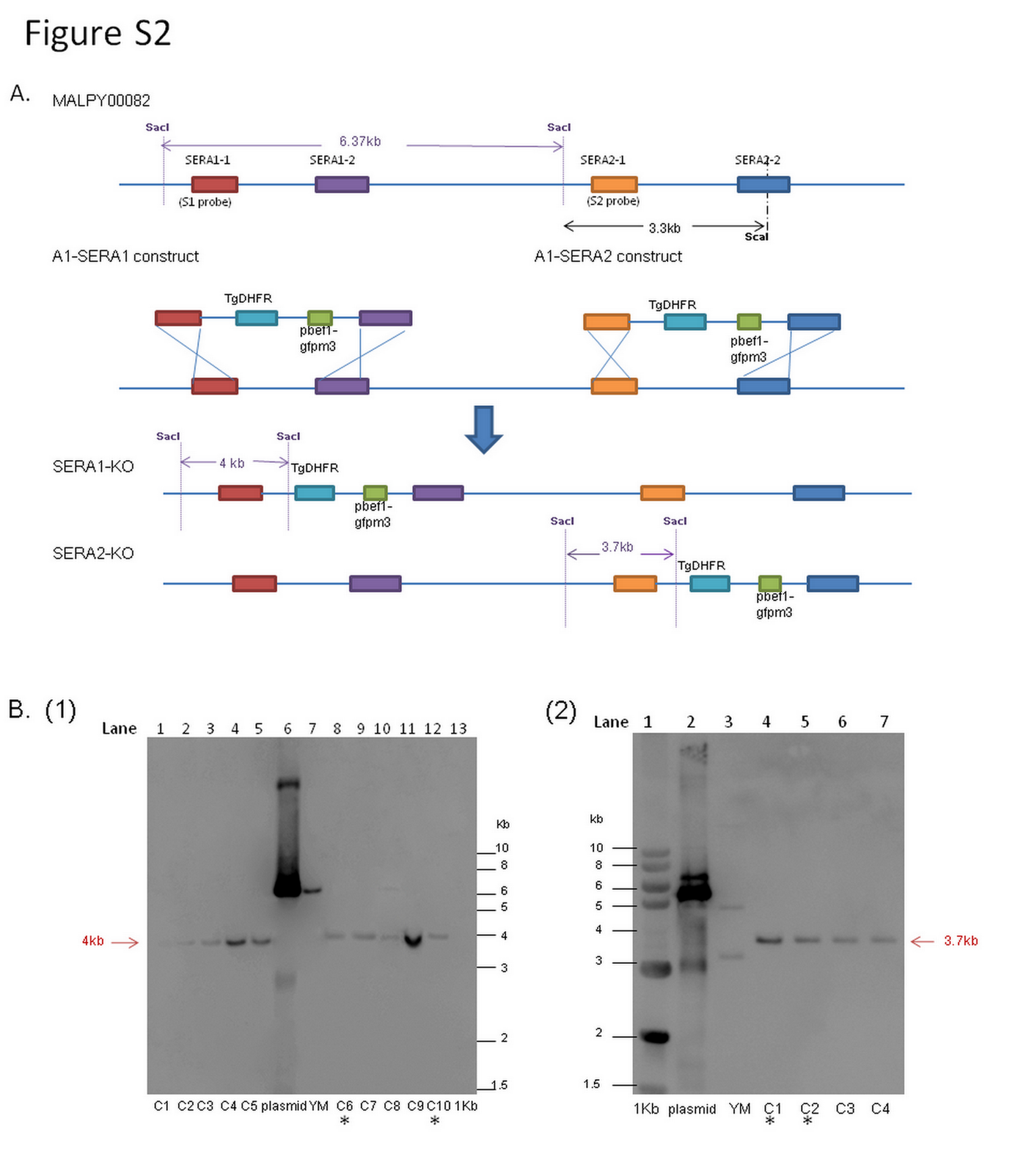

Supplement: Figure S2 — Disruption of SERA1 or SERA2 using homologous recombination. A- Genomic locus MALPY00082 coding for SERA1 and SERA2 showing the regions (red and purple in SERA1, orange and blue in SERA2) used for targeting the locus by a double cross-over strategy. Homologous recombination with the linearized plasmid containing the selectable marker and a detection marker flanked by the targeting sequences results in the SERA1-KO locus or SERA2-KO locus. GFP driven by the constitute promoter pbef1 is used for primary selection by FACs sorting. Restriction sites used for Southern blot analysis as well as region used for Southern blot probes (S1 probe and S2 probe) are also indicated. B- Southern blot screening of parasites for correct integration. (1) SacI digested DNA obtained from wild type YM (lane7) and transfection plasmid (lane6) as well as transfected parasite lines by limiting dilution C1 to C10 (lane1–5 and lane8–12) was analyzed by Southern blot using a SERA1 specific probe (S1). The expected fragment of 4 kb can be seen in all obtained transfected parasite lines, C6 and C10 were selected for further analysis.(2) SacI/ScaI digested DNA obtained from YM (lane 3) and tansfection plasmid (lane2) as well as transfected parasite lines by limiting dilution C1 to C4 (lane4–7) was analyzed by Southern blot using a SERA2 specific probe (S2). A single band of the expected fragment of 3.7 kb can be seen in all obtained parasite lines, C1 and C2 were selected for further analysis. (TIF) [file pone.0060723.s002.tif]

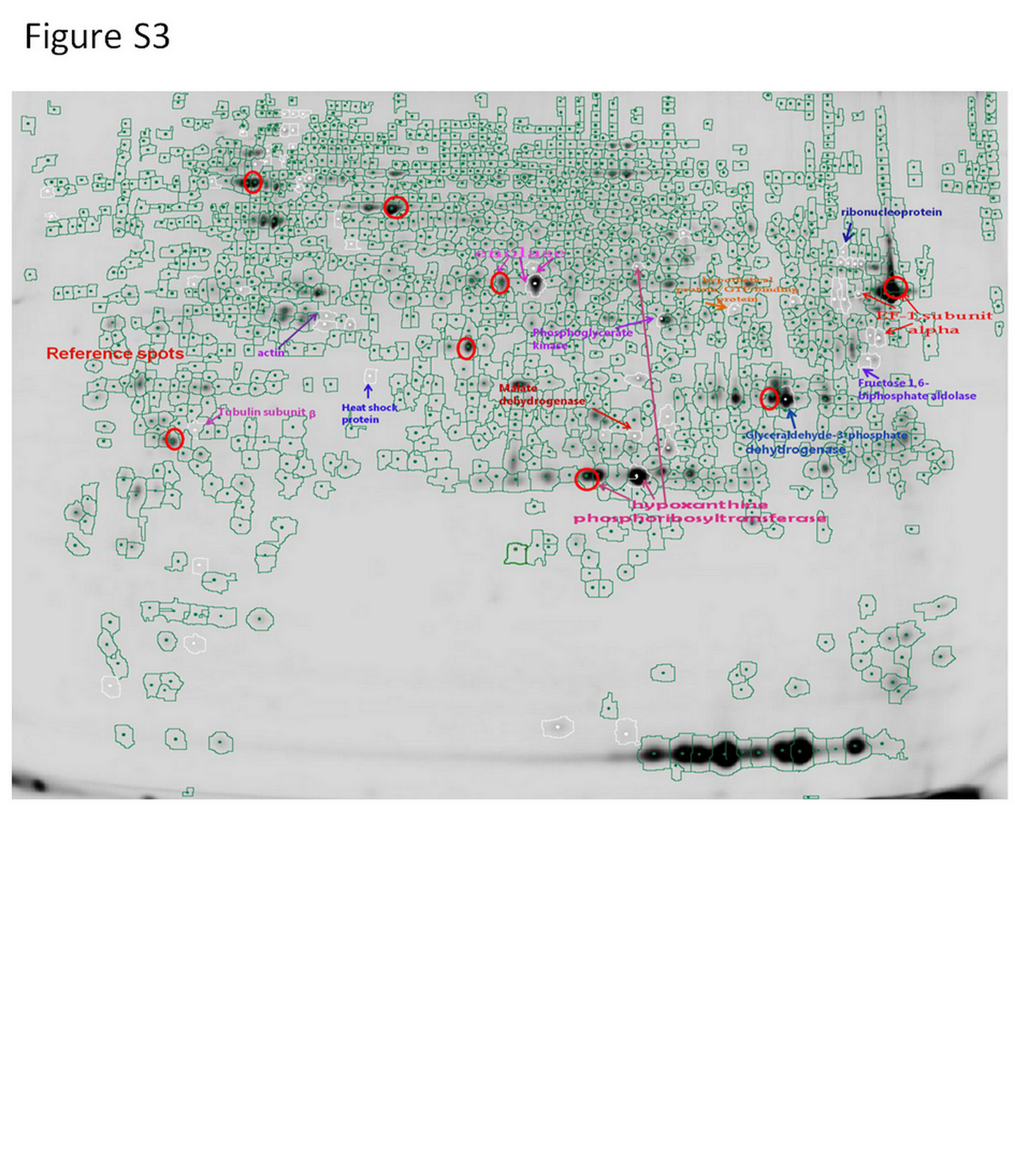

Supplement: Figure S3 — Representative two-dimensional DIGE gel of P.yoelii merozoite proteins comparison between wildtype YM and SERA2-ko with silver staining. Reference spots are marked with red circles, and spots for protein of interest selected for MALDI-TOF-TOF mass spectrometry are indicated. (TIF) [file pone.0060723.s003.tif]
