# Supplementary material for: The Role of Serine-Type Serine Repeat Antigen in Plasmodium yoelii Blood Stage Development
Source: PLoS One. 2013 Apr 25;8(4):e60723. doi: 10.1371/journal.pone.0060723 (PMC3636278; doi:10.1371/journal.pone.0060723)
Supplement: Table S1 — Primer pairs for generating southern blot probes. (DOCX) [file pone.0060723.s004.docx]

.

| Southern blot probe | Primer Sequence |
| --- | --- |
| S1 probe  (for SERA1) | Forward primer: 5’-CTGTACCGGGAAACTCTGATC-3’ |
|  | Reverse primer: 5’-ATCAGAATCAGATATGGTTGGC-3’ |
| S2 probe  (for SERA2) | Forward primer: 5’-TCTTCGGATTCTTCTGATGTAAC-3’ |
|  | Reverse primer: 5’-TAACGTTATTTTTAGAATCTACGC-3’ |
