# Supplementary material for: The Role of Serine-Type Serine Repeat Antigen in Plasmodium yoelii Blood Stage Development
Source: PLoS One. 2013 Apr 25;8(4):e60723. doi: 10.1371/journal.pone.0060723 (PMC3636278; doi:10.1371/journal.pone.0060723)
Supplement: Table S2 — PCR primers for eGFP-tagged SERA1 and SERA2 parasites determination. (DOCX) [file pone.0060723.s005.docx]

| PCR Primer Name | Primer Sequence |
| --- | --- |
| 291_5’intF | TTTGATGGAGTTATAGATTTACCTTTACC |
| 291_3’intR | GCAGTTTGACATTCACTCCATTTCG |
| 292_5’intF | TTGTCAATAGAAAGGGTTTGAAATTACC |
| 292_3’intR | CCATCTTGACTTGATCCTCCTCCAC |
| eGFP_5’intR | TTCAGCTCGATGCGGTTCACC |
| Rap2/3_3’intF | TGAGAAGAATGATTAAAGATCCCG |
